# COVID-19 Outcomes Amongst Patients With Pre-existing Cardiovascular Disease and Hypertension

**DOI:** 10.7759/cureus.13420

**Published:** 2021-02-18

**Authors:** Raja Chandra Chakinala, Chail D Shah, Jigisha H Rakholiya, Mehwish Martin, Nirmaljot Kaur, Harmandeep Singh, Toochukwu L Okafor, Chika Nwodika, Payu Raval, Salma Yousuf, Komal Lakhani, Angelina Yogarajah, Preeti Malik, Jagmeet Singh, Asim Kichloo, Urvish K Patel

**Affiliations:** 1 Medicine, Geisinger Commonwealth School of Medicine, Danville, USA; 2 Medicine, Guthrie Robert Packer Hospital, Sayre, USA; 3 Medicine, Mahatma Gandhi Medical College and Research Institute, Navi Mumbai, IND; 4 Internal Medicine, Mayo Clinic, Rochester, USA; 5 Public Health, Icahn School of Medicine at Mount Sinai, New York, USA; 6 Internal Medicine, Sri Guru Ramdas Institute of Medical Sciences and Research, Amritsar, IND; 7 Internal Medicine, Sri Guru Ramdas University of Health Sciences, Amritsar, IND; 8 Internal Medicine, Larkin Community Hospital, Hialeah, USA; 9 Internal Medicine, Oba Okunade Sijuwade College of Medicine, Igbinedion University, Okada, NGA; 10 Internal Medicine, siParadigm Diagnostic Informatics, Pine Brook, USA; 11 Internal Medicine, Lenox Hill Hospital, New York, USA; 12 Internal Medicine, Medical University of the Americas, Devens, USA; 13 Neurology, Massachusetts General Hospital, Andover, USA; 14 Nephrology, Geisinger Commonwealth School of Medicine, Scranton, USA; 15 Internal Medicine, Central Michigan University, Saginaw, USA; 16 Public Health and Neurology, Icahn School of Medicine at Mount Sinai, New York, USA

**Keywords:** coronavirus disease 2019 (covid-19), novel coronavirus disease 2019, sars-cov-2, invasive mechanical ventilation, hypertension, cardiovascular disease, systematic review and meta-analysis, 2019-ncov, atrial fibrillation, risk factors

## Abstract

Introduction: Coronavirus disease 2019 (COVID-19) has multiorgan involvement and its severity varies with the presence of pre-existing risk factors like cardiovascular disease (CVD) and hypertension (HTN). Therefore, it is important to evaluate their effect on outcomes of COVID-19 patients. The objective of this meta-analysis and meta-regression is to evaluate outcomes of COVID-19 amongst patients with CVD and HTN.

Methods: English full-text observational studies having data on epidemiological characteristics of patients with COVID-19 were identified searching PubMed from December 1, 2019, to July 31, 2020, following Meta-analysis Of Observational Studies in Epidemiology (MOOSE) protocol. Studies having pre-existing CVD and HTN data that described outcomes including mortality and invasive mechanical ventilation (IMV) utilization were selected. Using random-effects models, risk of composite poor outcomes (meta-analysis) and isolated mortality and IMV utilization (meta-regression) were evaluated. Pooled prevalence of CVD and HTN, correlation coefficient (r) and odds ratio (OR) were estimated. The forest plots and correlation plots were created using random-effects models.

Results: Out of 29 studies (n=27,950) that met the criteria, 28 and 27 studies had data on CVD and HTN, respectively. Pooled prevalence of CVD was 18.2% and HTN was 32.7%. In meta-analysis, CVD (OR: 3.36; 95% CI: 2.29-4.94) and HTN (OR: 1.94; 95% CI: 1.57-2.40) were associated with composite poor outcome. In age-adjusted meta-regression, pre-existing CVD was having significantly higher correlation of IMV utilization (r: 0.28; OR: 1.3; 95% CI: 1.1-1.6) without having any association with mortality (r: -0.01; OR: 0.9; 95% CI: 0.9-1.1) among COVID-19 hospitalizations. HTN was neither correlated with higher IMV utilization (r: 0.01; OR: 1.0; 95% CI: 0.9-1.1) nor correlated with higher mortality (r: 0.001; OR: 1.0; 95% CI: 0.9-1.1).

Conclusion: In age-adjusted analysis, though we identified pre-existing CVD as a risk factor for higher utilization of mechanical ventilation, pre-existing CVD and HTN had no independent role in increasing mortality.

## Introduction

In early December 2019, a large number of pneumonia cases were diagnosed in Wuhan, Hubei, China. The disease was later named as coronavirus disease 2019 (COVID-19), and the causative agent is severe acute respiratory syndrome coronavirus 2 (SARS-CoV-2) [1]. As of December, 2020, 69 million cases have been confirmed and 1.5 million people have died worldwide due to COVID-19 [2]. Common symptoms include fever, dry cough, fatigue, anorexia, myalgia and dyspnea [3]. In addition to the common symptoms, it is essential to know the impact of patients' comorbidities including cardiovascular disease (CVD) and hypertension (HTN) on COVID-19 outcomes. Renin-angiotensin-aldosterone system may play an important role in the pathogenesis of COVID-19 infection. Seventy-six percent of amino acid sequence of the spike protein of SARS-CoV-2 is similar to SARS-CoV [4]. SARS-CoV-2 affects binding of human cells to angiotensin-converting enzyme 2 (ACE2) an enzyme cleaving angiotensin II into angiotensin 1-7 [5]. SARS virus enters the cells via contact between the ACE2 receptor and the spike protein. SARS-CoV-2 may have more affinity towards ACE2 receptors in vitro [6].

According to a study performed in Brescia, Lombardy, Italy, patients with preexisting cardiovascular disease have poor prognosis (higher mortality, higher thromboembolic events and septic shock rates) compared to the patients without preexisting CVD [7]. COVID-19 usually results in mild symptoms but in some of infected patients, it can cause severe cardiac and lung disease [8]. Patients with CVD, especially heart failure, have a more severe clinical course once they are infected. The myocardial injury, which is demonstrated by an elevation in the troponin levels, has been noticed in at least 10% of hospitalized COVID-19 patients with a much higher percentage (25%-35%) noted when patients are severely ill with co-morbid CVD [5].

A study conducted by Guan et al. highlights that the patients with COVID-19 who develop severe pneumonia had higher rates of pre-existing HTN, diabetes, and CVD as compared to those who develop non-severe pneumonia. Patients with the above-listed cardiovascular co-morbidities also had an increased rate of admission to an intensive care unit (ICU), utilization of invasive mechanical ventilation (IMV) or death. Hence, patients with pre-existing CVD and CV risk factors appear to develop a worse disease outcome [3]. Information regarding the impact of patients' comorbidities, including CVD and HTN, on COVID-19 outcomes is limited.

In this article, we carried out a pooled analysis of existing observational studies to identify the prevalence of CVD and HTN and outcomes in COVID-19 patients with pre-existing CVD and HTN.

## Materials and methods

Endpoints

The primary aim of this study was to evaluate the association between outcomes of COVID-19 patients with pre-existing CVD and HTN. The secondary aim of this study was to identify the pooled prevalence of CVD and HTN among COVID-19 hospitalized patients. We have considered all-cause in-hospital mortality and mechanical ventilation utilization as our outcomes. Pre-existing CVD is defined as a history of coronary artery disease, history of percutaneous coronary intervention (PCI), coronary artery bypass grafting (CABG), atrial fibrillation, valvular heart disease, and heart failure. A composite poor outcome was defined by ICU admission, oxygen saturation (SpO_2_) <90%, IMV utilization, severe disease and in-hospital mortality. Severe disease was defined by respiratory distress, respiratory rate ≥30 breaths/min with SpO_2_ ≤93% at rest, PaO_2_/FIO_2_ ≤300, patients with >50% lung lesion progression within 24 to 48 hours, and respiratory failure requiring mechanical ventilation, shock, and organ failure requiring ICU treatment.

Search strategy and selection criteria

**Figure 1:**
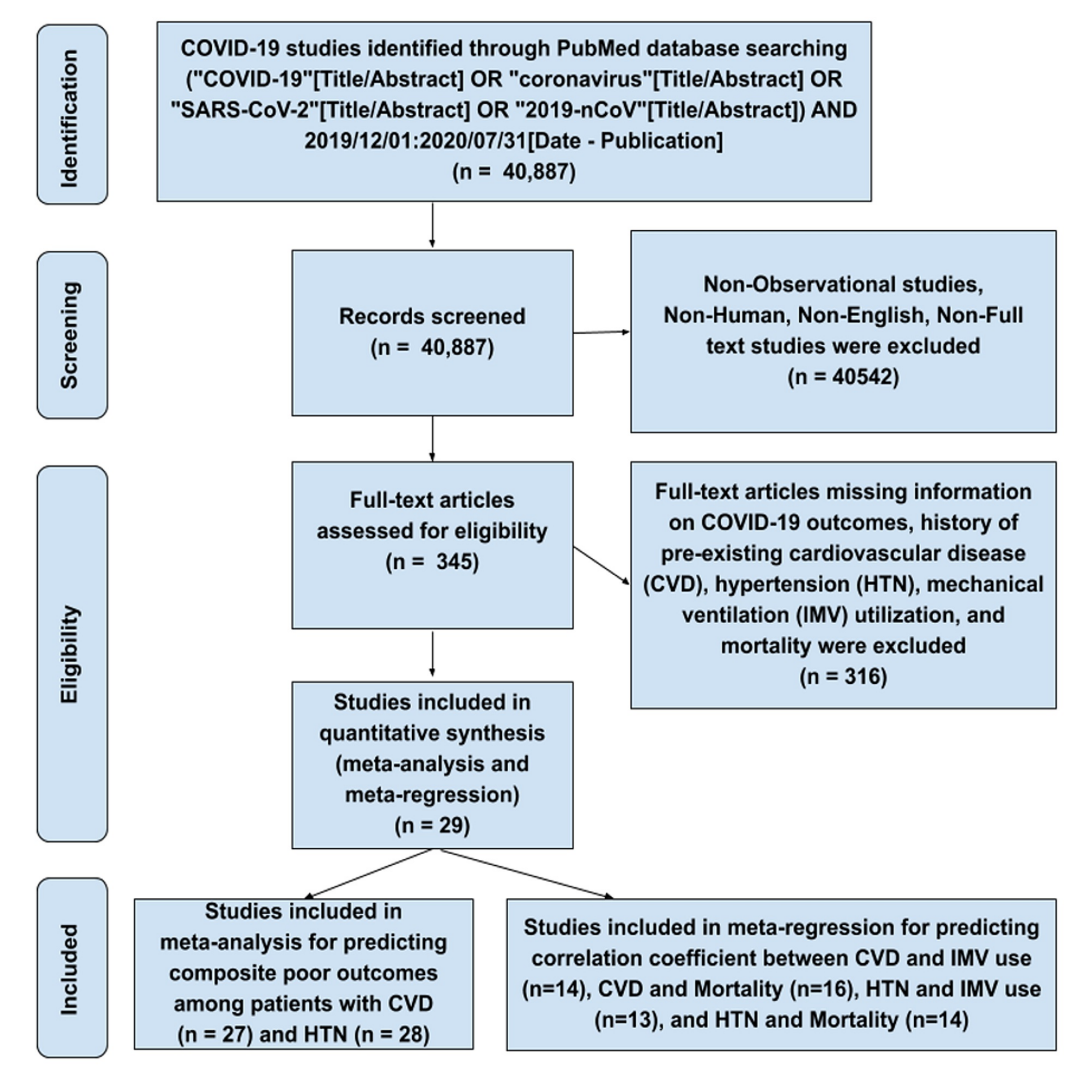
Flow diagram describing the study selection process COVID-19, coronavirus disease 2019; SARS-CoV-2, severe acute respiratory syndrome coronavirus 2; IMV, invasive mechanical ventilation

A systematic review and meta-analysis were performed using Meta-analysis Of Observational Studies in EpidemiologyMeta-analysis Of Observational Studies in Epidemiology (MOOSE) guidelines and Preferred Reporting Items for Systematic Reviews and Meta-analyses (PRISMA) protocol. We searched PubMed for observational studies that described characteristics of COVID-19 from December 1, 2019, to July 31, 2020, following query: ("COVID-19"[Title/Abstract] OR "coronavirus"[Title/Abstract] OR "SARS-CoV-2"[Title/Abstract] OR "2019-nCoV"[Title/Abstract]) AND 2019/12/01:2020/07/31[Date - Publication]. Studies describing the epidemiology of COVID-19 were included. Literature other than observational studies, non-English literature, non-full text, and animal studies were excluded. A flow diagram of literature search and study selection process is described in Figure 1.

Study selection

Abstracts and full-length articles were reviewed for availability of data on outcomes and pre-existing CVD and HTN as comorbidity for quantitative analysis. CS, JR, MM, and TO screened all identified studies and assessed full-texts to decide eligibility. Any disagreement was resolved through discussion with other reviewers (NK and HS).

Data collection

From the included studies, data relating to patient age and history of HTN and CVD were gathered on collection forms by three authors (CN, PR, SY), with a common consensus of two authors (KL, AY) upon disagreement. Details on outcomes such as mortality and needs for IMV were collected using pre-specified data collection templates. The study characteristics like publication year, country of origin, and sample size, study design, mean age of patients, and type of adverse outcomes are described in Table 1, with included studies and study data collected as a part of Malik et al.'s work and following similar methods and definition of adverse outcomes [9].

**Table 1:** Characteristics of the studies considered for the review CVD, cardiovascular disease; HTN, hypertension *Definition of pre-existing CVD was not specified in most of the studies, but mainly covered coronary artery disease, history of percutaneous coronary intervention, coronary artery bypass grafting, atrial fibrillation, valvular heart disease, and heart failure. ^a^Severe disease is defined by respiratory distress, respiratory rate ≥30 breaths/min with SpO_2_ ≤93% at rest, PaO_2_/FIO_2_ ≤300, patients with >50% lesion progression within 24 to 48 hours, and respiratory failure requiring mechanical ventilation, shock, and other organ failure requiring ICU treatment.

Study	Country	Sample size (n)	Study design	Mean age (years)	Adverse outcomes	Pre-existing CVD* and HTN
Huang et al., 2020	China	41	Prospective single-center	49	ICU admission	Pre-existing CVD (afibrillation) and HTN
Guan et al., 2020	China	1099	Retrospective multi-center	47	ICU admission/mechanical ventilation/death	Pre-existing CVD and HTN
Zheng et al., 2020	China	34	Retrospective single-center	66	Invasive mechanical ventilation	Pre-existing CVD and HTN
Wang et al., 2020	China	138	Retrospective single-center	56	ICU admission	Pre-existing CVD and HTN
Chen et al., 2020	China	21	Retrospective single-center	56	Severe disease^a^	HTN
Hong et al., 2020	South Korea	98	Retrospective single-center	55	ICU admission	Pre-existing CVD and HTN
Wang et al., 2020	China	69	Retrospective single-center	42	SpO_2_<90%	Pre-existing CVD and HTN
Mo et al., 2020	China	155	Retrospective single-center	54	Refractory pneumonia	Pre-existing CVD and HTN
Wu et al., 2020	China	201	Retrospective single-center	51	ARDS	Pre-existing CVD and HTN
Zhou et al., 2020	China	191	Retrospective multi-center	56	Death	Pre-existing CVD and HTN
Wang et al., 2020	China	339	Retrospective single-center	69	Death	Pre-existing CVD and HTN
Huang et al., 2020	China	202	Retrospective multi-center	44	Severe disease^a^	Pre-existing CVD and HTN
Colaneri et al., 2020	Italy	44	Retrospective single-center	67	Severe disease^a^	Pre-existing CVD and HTN
Goyal et al., 2020	USA	393	Retrospective multi-center	62.2	Invasive mechanical ventilation	Pre-existing CVD and HTN
Ruan et al., 2020	China	150	Retrospective multi-center	58.5	Death	Pre-existing CVD and HTN
Du R et al., 2020	China	179	Prospective single-center	57.6	Death	Pre-existing CVD and HTN
Qin et al., 2020	China	452	Retrospective single-center	58	Severe disease^a^	Pre-existing CVD and HTN
Paranjpe et al., 2020	USA	2199	Retrospective single-center	65	Death	Pre-existing CVD and HTN
Zheng et al., 2020	China	161	Retrospective single-center	45	Severe disease^a^	Pre-existing CVD and HTN
Zhang et al., 2020	China	663	Retrospective single-center	55.6	Death	Pre-existing CVD and HTN
Mikami et al., 2020	USA	6493	Retrospective multi-center	59	Death	HTN
Marcello et al., 2020	USA	13442	Retrospective multi-center	52.7	Death	Pre-existing CVD and HTN
Wang et al., 2020	China	275	Retrospective single-center	49	Severe disease^a^	Pre-existing CVD and HTN
Cao et al., 2020	China	80	Retrospective single-center	53	Severe disease^a^	Pre-existing CVD and HTN
Shahriarirad et al., 2020	Iran	113	Retrospective multi-center	53.75	Severe disease^a^	Pre-existing CVD and HTN
Suleyman et al., 2020	USA	463	Prospective multi-center	57.5	ICU admission	Pre-existing CVD and HTN
Khamis et al., 2020	Oman	63	Retrospective multi-center	48	ICU admission	Pre-existing CVD and HTN
Zhang et al., 2020	China	140	Retrospective single-center	57	Severe disease^a^	Pre-existing CVD and HTN
Yang et al., 2020	China	52	Retrospective single-center	59.7	Death	Pre-existing CVD
Total studies = 29		Total, n=27,950				

Statistical analysis

The pooled prevalences of pre-existing CVD and HTN were calculated. Meta-analysis was performed using Review Manager (Cochrane RevMan 5; Cochrane, London), and Maentel-Haenszel random-effects models were used to pool odds ratio (OR), 95%CI, p-value, and heterogeneity (I²) to evaluate the relationship between both comorbidities and composite poor outcomes/severe disease of COVID-19 patients in each study. The forest plots were created.

Age-adjusted meta-regression was performed using Comprehensive Meta-Analysis software (Biostat Inc., Englewood, NJ) used estimated correlation coefficient (r), odds ratios [e^ coefficient], 95% CI, p-value, and I^2^ between these comorbidities and IMV utilization and mortality. The correlation plots were created using random-effects models. A p-value of less than 0.05 was considered significant and I² >75% was considered significant heterogeneity.

## Results

Review of the databases identified 40,887 articles, out of which 345 full-text articles were assessed for eligibility using inclusion and exclusion criteria. During the second round, we excluded 316 articles with insufficient clinical information on COVID-19 outcomes. A total of 29 articles on COVID-19 and outcomes due to pre-existing CVD and HTN were extracted for final evaluation. After detailed assessment, as of July 31, 2020, we included 27 articles for meta-analysis for predicting composite poor outcomes among patients with CVD and 28 articles for HTN. For meta-regression to predict the correlation between CVD and IMV use, CVD and mortality, HTN and IMV use, and HTN and mortality, we considered 14, 16, 13 and 14 articles, respectively.

Pooled prevalence of CVD and HTN

The pooled prevalence of pre-existing CVD was 18.2% (2144/11,775 patients) and for HTN was 32.7% (4572/13,966 patients) amongst COVID-19 hospitalizations.

Meta-analysis showing prediction between composite poor outcomes (severe disease) and comorbidities (CVD and HTN)

**Figure 2:**
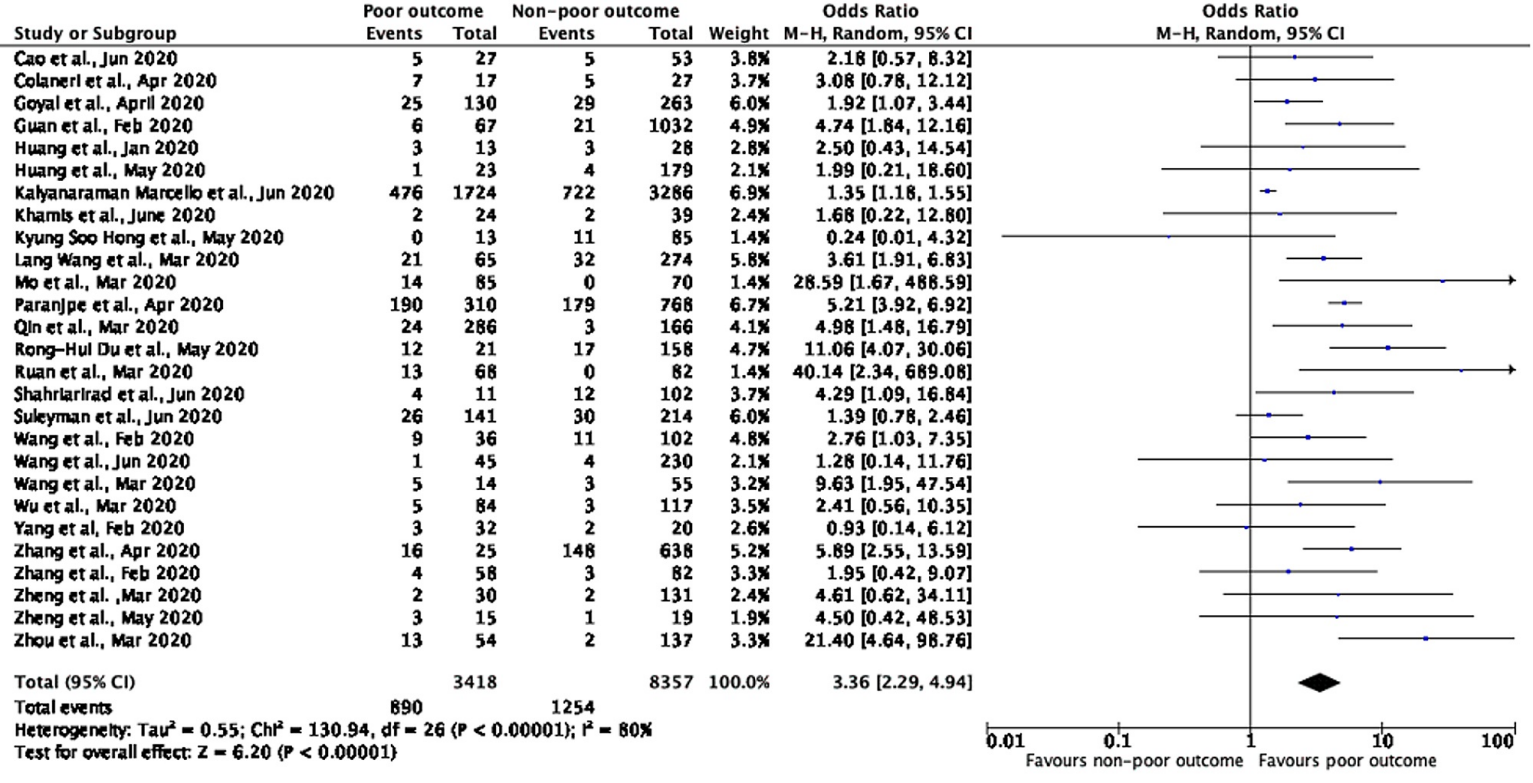
Meta-analysis showing prediction between composite poor outcomes and cardiovascular disease

A meta-analysis of 27 studies having details on CVD showed that patients with poor outcomes (severe disease) had higher odds of having pre-existing CVD compared to non-severe disease with a pooled OR of 3.36 (95% CI: 2.29-4.94; p<0.00001), with a significant between-study heterogeneity (p=0.00001; I²=80%) (Figure 2).

**Figure 3:**
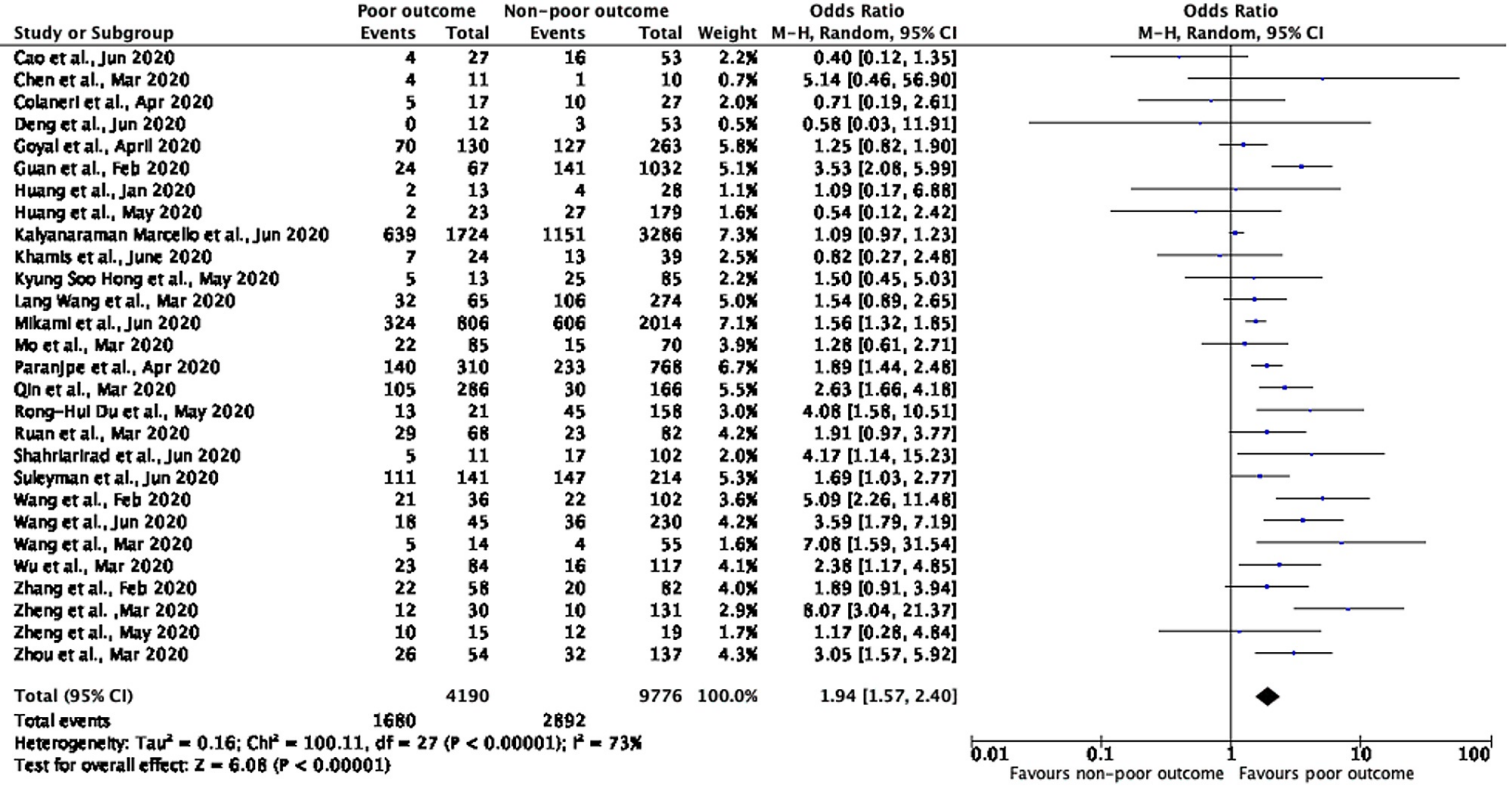
Meta-analysis showing prediction between composite poor outcomes and hypertension

A meta-analysis of 27 studies having details on HTN showed that patients with poor outcomes/severe disease had higher odds of having pre-existing HTN compared to non-severe disease with a pooled OR of 1.94 (95% CI: 1.57-2.40; p<0.00001), with a significant between-study heterogeneity (p=0.00001; I²=73%) (Figure 3).

Meta-regression showing correlation of pre-existing CVD with IMV utilization and mortality

**Figure 4:**
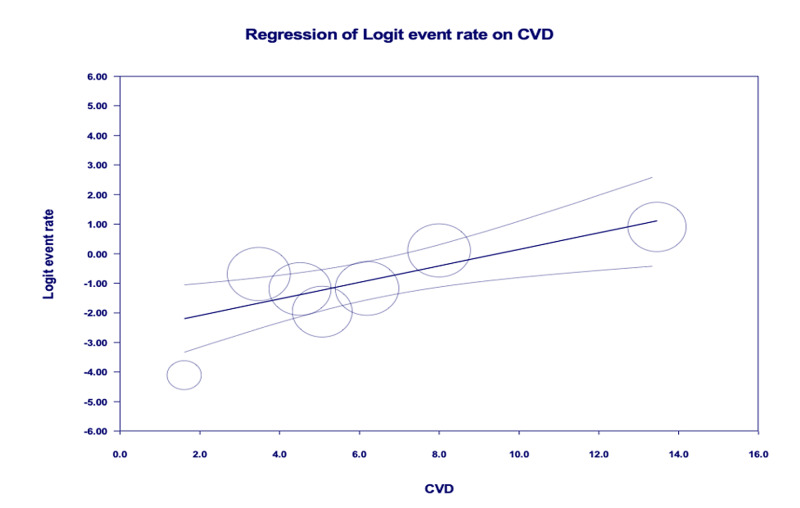
Age-adjusted meta-regression analysis for the evaluation of need for invasive mechanical ventilation amongst patients with CVD CVD, cardiovascular disease

**Figure 5:**
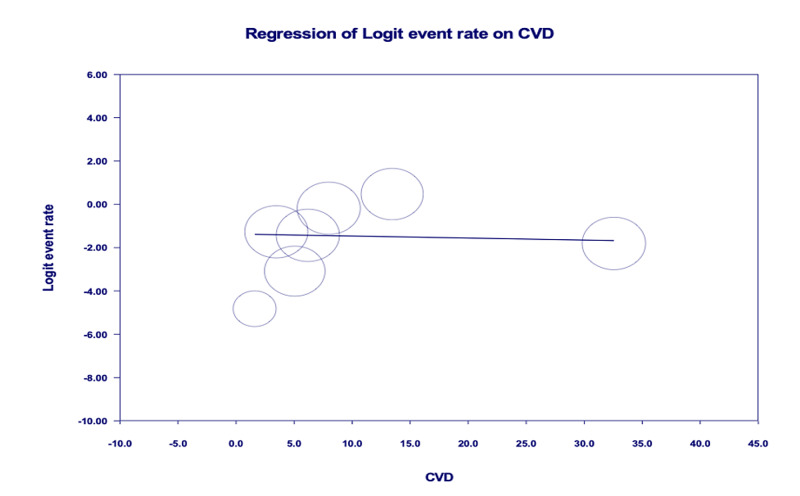
Age-adjusted meta-regression analysis for evaluation of mortality amongst patients with patients with CVD CVD, cardiovascular disease

Age-adjusted meta-regression analysis showed IMV utilization was significantly higher among COVID-19 patients with pre-existing CVD [r: 0.28; OR: 1.3 (1.1-1.6); I^2^: 89.7%; p=0.0028] (Figure 4). There was no significant correlation between pre-existing CVD and mortality [r: -0.01; OR: 0.9 (0.9-1.1); I^2^: 96.3%; p=0.8772] (Figure 5).

Meta-regression showing correlation of pre-existing HTN with IMV utilization and mortality

**Figure 6:**
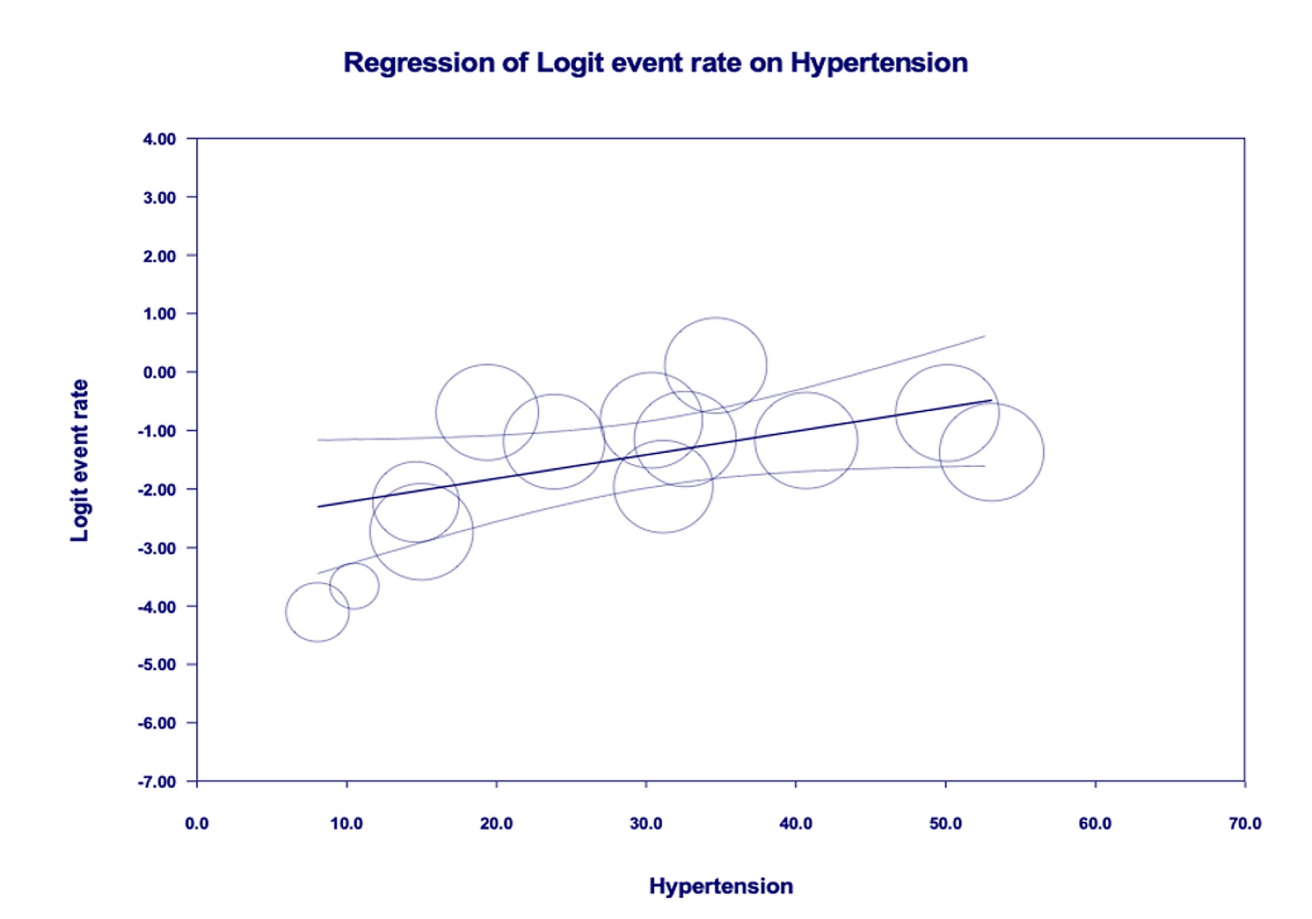
Age-adjusted meta-regression analysis for the evaluation of need for invasive mechanical ventilation amongst patients with hypertension

**Figure 7:**
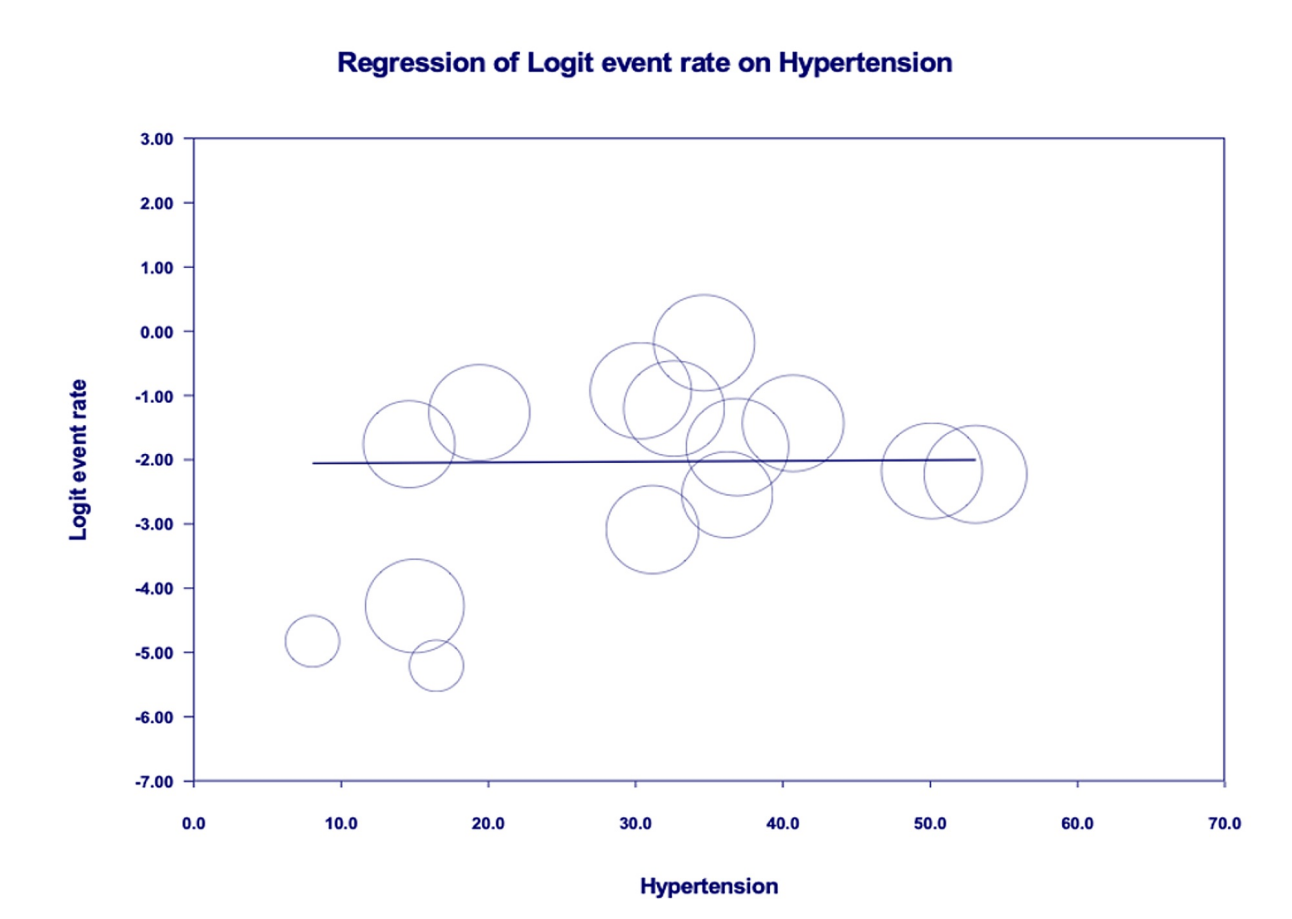
Age-adjusted meta-regression analysis for the evaluation of mortality amongst patients with hypertension

Age-adjusted meta-regression analysis showed HTN was neither significantly correlated with IMV utilization [r: 0.01; OR: 1.0 (0.9-1.1); I^2^: 95.9%; p=0.8161] (Figure 6) nor correlated with increased mortality [r: 0.001; OR: 1.0 (0.9-1.1); I^2^: 96%; p=0.9685] amongst COVID-19 patients (Figure 7).

Risk of bias assessment was performed using the Newcastle-Ottawa Scale and has shown that studies included in this meta-analysis have moderate to high risk of bias (Table 2).

**Table 2:** Risk of bias assessment using the Newcastle-Ottawa Scale

Study	Newcastle-Ottawa Scale			Overall risk of bias
	Selection	Comparability	Exposure	
Huang et al., 2020	***	*	*	Low
Guan et al., 2020	***	*	**	Moderate
Zheng et al., 2020	****	*	*	Low
Wang et al., 2020	***	*	**	Moderate
Chen et al., 2020	**	*	*	High
Hong et al., 2020	**	*	**	High
Wang et al., 2020	**	*	**	High
Mo et al., 2020	**	*	*	High
Wu et al., 2020	***	*	**	Moderate
Zhou et al., 2020	***	*	*	Moderate
Wang et al., 2020	***	*	**	Moderate
Huang et al., 2020	****	*	*	Low
Colaneri et al., 2020	***	*	**	Moderate
Goyal et al., 2020	**	*	*	High
Ruan et al., 2020	**	*	*	High
Rong-Hui Du et al., 2020	****	*	*	Low
Qin et al., 2020	***	*	**	Moderate
Paranjpe et al., 2020	****	*	*	Low
Zheng et al., 2020	***	*	**	Moderate
Zhang et al., 2020	***	*	*	Moderate
Mikami et al., 2020	***	*	**	Moderate
Marcello et al., 2020	***	*	**	Moderate
Wang et al., 2020	****	*	*	Low
Cao et al., 2020	**	*	*	High
Shahriarirad et al., 2020	***	*	*	Moderate
Suleyman et al., 2020	**	*	*	High
Khamis et al., 2020	****	*	*	Low
Zhang et al., 2020	***	*	*	Moderate
Yang et al., 2020	***	*	*	Moderate

## Discussion

The included studies have shown that vascular risk factors like pre-existing CVD, HTN, cerebrovascular disease, obesity, smoking, and diabetes were highly prevalent [10] and also associated with severe COVID-19 [11-15]. In our study, the prevalence of CVD is 18.2% and HTN is 32.7%. According to Matsushita et al., age is an independent risk factor for the severity [16], so we have adjusted our models for the age before predicting the disease severity. Our meta-regression showed patients with pre-existing CVD had higher odds of composite poor outcome (OR: 3.36) and age-adjusted meta-regression analysis showed pre-existing CVD had higher odds of need for IMV by 30% without significant relationship with high mortality. A very early report of 44,672 confirmed cases from China showed that the case-fatality rate was higher amongst pre-existing CVD, HTN (10.5% vs 6.0%) cases compared to overall case-fatality rate (2.3%) [17].

Potential mechanisms of myocardial injury in COVID-19 include hypoxia-induced myocyte damage and an immune-mediated cytokine storm. We know that SARS-CoV-2 tends to bind to the ACE2 expressed in the epithelial linings of the lungs, causing the COVID-19 disease. Since hypertensive patients are treated with ACE inhibitors or angiotensin receptor blockers, there has been a concern if the SARS-CoV-2 has any significant poor outcomes in such patients [5,18,19]. Patients with pre-existing CVD have a higher risk of developing acute cardiac injury and other cardiovascular complications. At regular intervals, cardiac troponin I and N-terminal pro-B-type natriuretic peptide (NT-proBNP) serve as important biomarkers that help to stratify patients according to their risk of cardiac injury and outcomes in hospitalized patients [20,21].

Our meta-analysis showed that COVID-19 patients with pre-existing HTN have poor outcomes. This was consistent with the retrospective study conducted in China where after adjusting for age and smoking status, hypertensive patients were more likely to reach the composite end-points (ICU admission, IMV utilization, or death) as compared to non-hypertensive patients with a hazard ratio of 1.58 [22]. One study showed that the OR of death is greater than 1 in patients with HTN with the greatest OR of 3.5 in patients with heart disease [23]. While as per our study, there is no significant association between HTN and mortality, Pranata et al. and Lippi et al. demonstrated that there is a higher risk of mortality in COVID-19 patients with HTN [12,24]. It was noted that there is no significant association of HTN with mortality and IMV utilization. Similar findings were inferred by a study in New York City including 5700 patients [25]. The discrepancy between other study findings and our meta-analysis results is explained by bigger sample size (n=27,950) pooled from 29 studies all over the world, high power, and performing age-adjusted analysis.

Strengths and limitations

To our knowledge, this is the one of the large meta-analyses showing meta-regression of 29 studies (USA and non-USA) that tried to demonstrate the association of HTN and pre-existing CVD with poor outcomes (ICU admisssions, needs for mechanical ventilator, or death). Our study has high heterogeneity. Studies are also missing details on the severity of these risk factors. We have not adjusted our results with others comorbidities and confounders. The long-term follow-up was missing amongst studies we have chosen for our analysis.

## Conclusions

Our findings may provide insights into designing models for the early identification of high-risk patients and prioritizing their treatment based on disease severity. More prospective studies should be planned to evaluate the role of concurrent comorbidities with CVD and HTN and composite effects of these comorbidities on outcomes of COVID-19 hospitalizations.
